# *Agaricus Blazei* Hot Water Extract Shows Anti Quorum Sensing Activity in the Nosocomial Human Pathogen *Pseudomonas Aeruginosa*

**DOI:** 10.3390/molecules19044189

**Published:** 2014-04-03

**Authors:** Marina Soković, Ana Ćirić, Jasmina Glamočlija, Miloš Nikolić, Leo J. L. D. van Griensven

**Affiliations:** 1 Department of Plant Physiology, Institute for Biological Research “Siniša Stanković”, University of Belgrade, Bulevar Despota Stefana 142, Belgrade 11000, Serbia; 2 Plant Research International, Wageningen University and Research Centre; Droevendaalsesteeg 1, Wageningen 6700 AA, The Netherlands

**Keywords:** *Agaricus blazei*, mushroom, antiqourum sensing activity, antimicrobial activity, antibiofilm activity, *Pseudomonas aeruginosa*

## Abstract

The edible mushroom *Agaricus blazei* Murill is known to induce protective immunomodulatory action against a variety of infectious diseases. In the present study we report potential anti-quorum sensing properties of *A. blazei* hot water extract. Quorum sensing (QS) plays an important role in virulence, biofilm formation and survival of many pathogenic bacteria, including the Gram negative *Pseudomonas aeruginosa*, and is considered as a novel and promising target for anti-infectious agents. In this study, the effect of the sub-MICs of *Agaricus blazei* water extract on QS regulated virulence factors and biofilm formation was evaluated against *P. aeruginosa* PAO1. Sub-MIC concentrations of the extract which did not kill *P. aeruginosa* nor inhibited its growth, demonstrated a statistically significant reduction of virulence factors of *P. aeruginosa*, such as pyocyanin production, twitching and swimming motility. The biofilm forming capability of *P. aeruginosa* was also reduced in a concentration-dependent manner at sub-MIC values. Water extract of *A. blazei* is a promising source of antiquorum sensing and antibacterial compounds.

## 1. Introduction

In recent years a growing interest has developed in the mechanisms of action of medicinal mushrooms. For over a thousand years mushrooms have been used in folk medicine in Asia to prevent and cure a multitude of diseases of quite different nature. The most well known examples are *Ganoderma lucidum*, *Phellinus linteus*, *Cordyceps sinensis*, *Trametes versicolor* and *Inonotus obliquus*. *Agaricus blazei* Murill (ABM) is a relative newcomer that was first found in 1960 in Piedade (Brazil) by Takatoshi Furumoto as “Cogumelo do sol” and later identified by Heinemann [1]. Its formal name is now *A. brasiliensis* [2], although it is usually called *A. blazei* Murill. 

Mostly based on *in vitro* studies the medicinal effects of ABM have been attributed to β-glucans, phenols and terpenes [3,4]. The β-glucans are immunomodulators, and phenols and terpenes are redox regulators; both enhance or attenuate the immune system of higher animals by inducing or inhibiting the production of pro- and anti-inflammatory cytokines [4,5]. *In vitro* ABM induces and balances immunity once pro-inflammatory processes are taking place [6].

In higher animals ABM quickly stimulates innate immunity, *i.e.*, causes rapid pro-inflammatory effects when under threat by infectious agents or cancer [3,4,5,6]. The antimicrobial effects of ABM such as against *Mycobacterium tuberculosis*, *M. bovis* and *Streptococcus pneumoniae* have been attributed to the immune system [5,7]. The preventive role of innate immunity was contradicted by the study of Fantuzzi *et al.* [8] who showed that ABM extract did not promote immunostimulation and protection during experimental *Salmonella enterica* infection in mice. 

Many pathogens use the formation of biofilms as a defense against their host’s immune system and against antibiotic treatment. Biofilms are vast bacterial populations in a host that are protected by a layer of polymeric substances [9]. Biofilms use quorum sensing for their protection, a bacterial coordination system that allows density-dependent cell–cell communication, in which small diffusible signalling molecules globally regulate expression of various genes including antibiotic resistance swarming motility, exopolysaccharide production, virulence, and cell aggregation [10,11].

It is important to emphasize that of all the infectious diseases, at least 65% are associated with the bacterial communities which proliferate by forming biofilms [9,10,11]. Inner-ear infections in children, gingivitis, urinary tract infections, dental plaque and chronic wounds are all relatively insensitive to common antibiotic treatment because of biofilm formation and quorum sensing [10,11].

*Pseudomonas aeruginosa*, as an example, is an opportunistic human pathogen that infects immunocompromised individuals and people with cystic fibrosis [12]. It is an asporogenous, Gram negative, aerobic bacillus which is commonly found in the intestinal tract of higher animals, in sewage and in soil. It is a major cause of nosocomial infections and spreads easily through contaminated equipment and lack of hygiene. *P. aeruginosa* can grow within a host without harming it, until a threshold concentration is reached. Then they become aggressive, developing to the point at which their numbers are sufficient to overcome the host’s immune system. *P. aeruginosa* constitute a major risk for immunologically compromised patients and is involved in the etiology of bronchopneumonia, septic shock and wound infections [9]. *P. aeruginosa* have low sensitivity to antibiotic treatment, and have become multidrug resistant, which causes an increasing public health threat [13,14,15]. No new broad spectrum antibiotics have been developed recently for most Gram negative bacteria.

The worldwide emergence of higher level tolerance of target organisms against available broad spectrum antibiotics is a pressing global public health problem [16]. Considering the rapid spread of multidrug resistance, the development of new antimicrobial or antivirulence agents that act upon newly adapted microbial targets has become a very pressing priority [17]. Research efforts have focused on natural products which might be nontoxic inhibitors of quorum sensing [18,19], thus controlling infections without encouraging the appearance of resistant bacterial strains [20].

Recently we have described the direct antibiotic effects of ABM extracts against various bacteria (unpublished data) [21]. MIC’s and MBC’s of these extracts turned out equal to or better for inactivation of *P. aeruginosa* than those of ampicillin and streptomycin. The present study describes a search for anti-quorum sensing properties of the same extracts.

## 2. Results and Discussion

*A. blazei* mushrooms serve not only as a valued gourmet food, but are also used as medicinals and as food supplements to induce immune activity against a variety of infectious diseases, hepatitis and malignancies [7]. To our knowledge no direct antibiotic effects, *i.e.*, killing or inhibiting growth of microorganisms, have been reported for *A. blazei* until now.

Although considerable efforts have been done to define plant-derived natural products as sources of anti-quorum sensing compounds [22], no systematic search has been done among the kingdom of fungi. The only reports so far of possible anti-QS activity are from two plant root-associated fungi, *i.e.*, *Phialocephala fortinii* and *Meliniomyces variabilis* [23] that are able to interfere with the N-acylated homoserine lactone regulatory system of several Gram-negative bacteria, and of *Phellinus igniarius* [24] and *Ganoderma lucidum* [25] that can suppress violacein production of *Chromobacter violaceum*.

The present study connects the anti-QS properties of the popular medicinal mushroom *Agaricus blazei* Murill with the suppression of possible effects of a leading human pathogen, *i.e.*, *Pseudomonas aeruginosa*, which has become multi drug resistant. Unlike antibiotics anti-QS agents do not kill or inhibit bacterial growth, they just weaken their activity. Anti-QS effects are therefore visibly different from antibiotic effects in bacterial cultures on agar in that they do not create clear zones that are free of bacterial growth, but rather hazy zones.

The minimal inhibitory concentration (MIC) of *A. blazei* extract for *P. aeruginosa* was determined by the microdilution method to be 0.2 mg/mL in our previous study [21, unpublished data]. The effect on biofilm formation of *P. aeruginosa* was tested at lower values than the MIC, *i.e.*, with 0.1 (0.5 MIC), 0.05 (0.25 MIC) and 0.025 mg/mL (0.125 MIC). A concentration of 0.1 mg/mL of *A. blazei* extractallowed 16.06% biofilm formation, ampicillin 69.16% and streptomycin 49.40% in comparison with the nontreated control (98.90%). *A. blazei* extract at 0.05 mg/mL allowed biofilm formation of 42.58%, ampicillin 56.46% and streptomycin 70.97% in comparison with the control (100%). The lowest concentration of *A. blazei* extract (0.025 mg/mL) showed biofilm formation of 98.37%, ampicillin 92.16% and streptomycin 88.36% in comparison to the control value of 99.30%. Table 1 shows that *A. blazei* water extract reduced biofilm formation much more effectively than streptomycin or ampicillin.

**Table 1:** Effects of *Agaricus blazei* water extract on biofilm formation of *Pseudomonas aeruginosa* (PAO1). Mean ± SD diameters of the growth clear inhibition zones around the discs in millimeters.

Concentration	0.1 mg/mL *	0.05 mg/mL	0.025 mg/mL
***A. blazei* extract**	16.06 ± 0.47	42.58 ± 0.35	98.37 ± 0.97
**Ampicillin**	69.16 ± 0.65	56.46 ± 0.46	92.16 ± 0.37
**Streptomycin**	49.40 ± 0.46	70.97 ± 0.36	88.36 ± 0.42
***P. aeruginosa* control**	98.90 ± 0.97	100.00 ± 0.00	99.30 ± 0.46

* *A. blazei* extract at 0.1 mg/mL equals 0.5 MIC.

It can be seen that in the tested concentration only streptomycin showed inhibition zones at 0.125 mg/disc (9.0 mm), 0.25 mg/disc (16.6 mm) and 0.5 mg/disc (18.6 mm), while *A. blazei* extract and ampicillin possessed antiquorum sensing activity. Extract exhibited AQ effect at concentrations 0.03–0.5 mg/disc in range of 7.0–17.7 mm, and ampicillin possessed AQ activity (0.25–0.5 mg/disc) in the range of 7.6–9.0 mm as transparent zones around discs. The aqueous extract of *A. blazei* showed AQ effect in all tested concentrations (Table 2). According to our knowledge there are no published data about antiquorum sensing activity of extracts of *A. blazei*.

**Table 2:** Minimal inhibitory (MIC) and antiquorum (AQ) zones in mm induced by *Agaricus blazei* extract in the disc-diffusion method (mean ± SD).

Concentration mg/disc		0.03	0.06	0.125	0.25	0.50
***A. blazei* extr.**	**MIC**	-	-	-	-	-
	**AQ**	7.0 ± 1.0	7.3 ± 0.5	8.0 ± 1.0	9.3 ± 1.0	17.7 ± 1.0
**Ampicillin**	**MIC**	-	-	-	-	-
	**AQ**	--	--	--	7.6 ± 0.6	9.0 ± 1.0
**Streptomycin**	**MIC**	-	-	9.0 ± 1.0 15.0 ± 2.1	16.6 ± 1.5 22.6 ± 2.3	18.6 ± 1.2 26.7 ± 1.2
	**AQ**					
***P. aeruginosa* Control**	**MIC AQ**	ni	ni	ni	ni	ni

-: no inhibitory activity; --: no AQ, ni: no activity (control).

The quorum-sensing inhibition zone (Table 2) occurred behind the margin of the inhibition zone. Our previous study with the same extract had demonstrated strong antibacterial activity, especially against Gram-negative bacteria ([21], unpublished). *A. blazei* has a long history of use in traditional medicine. Their antibacterial activity, the anti-quorum sensing activity and possible other mechanisms may be responsible for their therapeutic efficacies.

**Figure 1:**
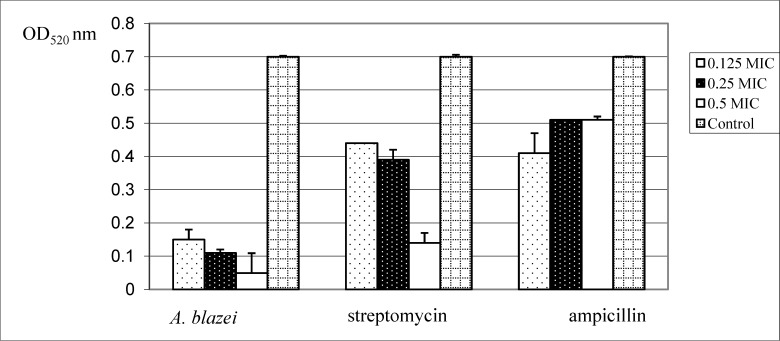
Reduction of pyocyanin pigment production of *Pseudomonas aeruginosa* by *Agaricus blazei* extract tested at subMIC, *i.e.*, 0.5 MIC (0.1 mg/mL), 0.25 MIC (0.05 mg/mL) and 0.125 MIC (0.025 mg/mL).

The activity against pyocyanin production in a flask assay was used to quantify quorum sensing inhibitory activity of the extracts. Pyocyanin is a green pigment which signals the upregulation of quorum sensing controlled genes during stationary growth of *P. aeruginosa* [26]. The *A. blazei* extract demonstrated concentration-dependent pyocyanin inhibitory activity. At all tested concentrations of the extract the green pigment content was decreased. The extract showed a stronger reduction of pigment than ampicillin and streptomycin (Figure 1). Promising anti-quorum sensing compounds have been demonstrated to disrupt bacterial biofilms and make the bacteria more susceptible to antibiotics, and these compounds also provide the ability to reduce bacterial virulence factors as well as promote clearance of bacteria in infectious animal models. Many mechanisms of action have been proposed to interfere with the quorum sensing system such as: (1) inhibition of biosynthesis of autoinducer molecules; (2) inactivation or degradation of the autoinducer; (3) interference with the signal receptor; and (4) inhibition of the genetic regulation system [27].

In addition to QS, the initiation of biofilm formation by *P. aeruginosa* depends on two cell-associated structures; the flagellum and type IV pili [28,29]. The flagellum is responsible for swimming motility while the type IV pili are responsible for twitching motility [30]. Both types of motility are important in the initial stages of biofilm formation by *P. aeruginosa* [28,29]. Therefore, we tried to determine if our extract will influence one or both motilities. On swimming plates, the motile strain PAO1 was used as the 100% standard (control) for motility while the Petri dishes with the same strain plus *A. blazei* extracts were compared with the control.

**Figure 2:**
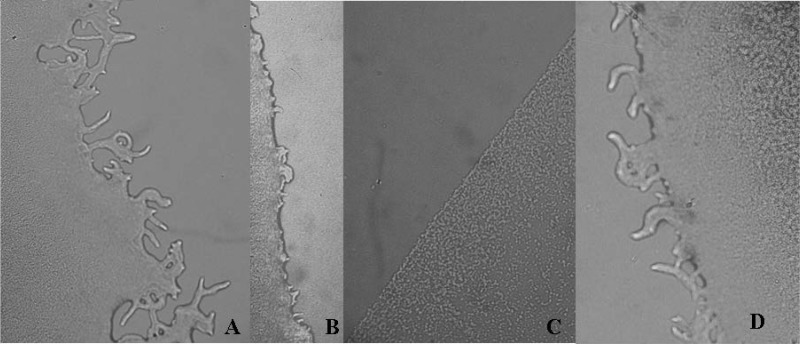
Flagella motility of *Pseudomonas aeruginosa* treated with *Agaricus blazei* extract.

The *A. blazei* hot water extract reduced the twitching motility of *P. aeruginosa*. The normal colonies of *P. aeruginosa*, *i.e.*, in the absence of the extract, were flat with a rough appearance displaying irregular colony edges (Figure 2A) and a hazy zone surrounding the colony. The cells were in a very thin layer. After 2 days of incubation at ambient temperature, colony expansion occurred very rapidly due to twitching motility, the control *P. aeruginosa* isolates produced swimming zones to 100% (Table 3) and was 14.0 mm. Bacteria that were grown with the *A. blazei* aqueous extract solution were incapable of producing such a twitching zone and had almost round, smooth, regular colony edges, the flagella were reduced both in size and in numbers, and the colony diameter swimming zone was not reduced (21.7 mm) (Figure 2B). Streptomycin reduced the flagellae completely (Figure 2C), while ampicillin did not affect the formation of flagella at all (Figure 2D, Table 3).

Light microscopy of colony edges of *P. aeruginosa* in twitching motility, grown in the presence or absence of *A blazei* extracts. *P. aeruginosa* produced a flat, widely spread, irregularly shaped colony in the absence of *A. blazei* extracts. *P. aeruginosa* produced a flat, widely spread, irregularly shaped colony in the absence of extracts (**A**); The colonies from the bacteria grown with *A. blazei* aqeous extract in concentration of 0.15 mg/mL (**B**) were rounded, had a smooth domed shape and lacked a hazy zone surrounding the colony. *P. aeruginosa* colony with streptomycin at a concentration of 0.15 mg/mL without flagella (**C**) and with ampicillin at 0.15 mg/mL with almost regularly formed flagella (**D**); Magnification: (**A**–**D**) × 100.

**Table 3:** Effect of *Agaricus blazei* extract on twitching activity, colony diameter and color of *Pseudomonas aeruginosa* (mean ± SD).

Extracts	Colony diameter	Colony color	Colony edge
*A. blazei* H_2_O	21.67 ± 0.51	white	reduced flagella
Streptomycin	5.00 ± 0.06	white	flat
Ampicillin	12.00 ± 1.00	white	regular
Control *P. aeruginosa* 10^9^ CFU	14.00 ± 1.00	green	regular

From the observed results it can be noticed that water extract of *A. blazei* exhibited antibacterial and also antiquorum activity. The extract showed effects on all tested mechanisms included in AQ; it had an influence on antibiofilm formation, exhibited antiquorum zones in the disc diffusion assay, also influenced flagella motility, reduced colony diameter and changed the color of the colony. Further it reduced pigment (pyocyanin) production by *P. aeruginosa.* Taken together this indicated that this extract could be used to prevent or/and to control *P. aeruginosa* growth.

It is interesting to notice that the microbial load of mushrooms, regardless of their origin (wild or cultivated), is dominated by the Gram-negative bacteria, pseudomonads and enterobacteriaceae [31]. *Pseudomonas sp.*, which cause drippy gill, mummification and brown discoloration (bacterial blotch) are able to completely destroy the cultivated button mushroom *Agaricus bisporus*. For *A. blazei* no reports have been published on possible infection by *Pseudomonas sp*. If anti-QS is causal in prevention of infection of *A. blazei* itself by *Pseudomonas sp.*, it would be worth trying to use the extract to prevent this disease in *A. bisporus*, which obviously lacks protection [32].

It is further interesting to note that most medicinal mushroom supplements on the market are crude hot water extracts. Their presumed protection against infection may not be caused by stimulation of innate immunity but rather by induction of anti-QS.

## 3. Experimental

### 3.1. Extract Preparation

Freshly cultivated *A. blazei* mushrooms (strain M7700, Mycelia bvb, Nevele, Belgium) were dried at 45 °C and then powdered in a Buhler type hammer mill equipped with a 1 mm sieve insert. For hot water extraction 50 grams of dry mushroom powder were homogenized in 1 L of water and extracted by autoclaving for 25 min at 121 °C. The resulting mass was centrifuged at 7.438× *g* for 30 min in a SLA-1500 rotor in a Dupont-Sorvall RC5C centrifuge (Newtown, CT, USA). The resulting supernatant was concentrated to 1/10 of its volume by evaporation and then lyophilized. The dry powder was stored dry and away from light for later use. The dry extract powder was solved in water (250 mg/mL) for further use.

### 3.2. Bacterial Strains, Growth Media and Culture Conditions

*P. aeruginosa* PA01 (ATCC 27853) used in this study is from the collection of the Mycoteca, Institute for Biological Research “Sinisa Stankovic”, Belgrade, Serbia. Bacteria were routinely grown in Luria-Bertani (LB) medium (1% *w/v* NaCl, 1% *w/v* tryptone, 0.5% *w/v* yeast extract) with shaking (220 rpm) and cultured at 37 °C.

### 3.3. Biofilm Formation

The effect of different concentrations of extract (ranging from 0.5 to 0.125 of MIC, MIC was 0.2 mg/mL) on biofilm forming ability was tested on polystyrene flat-bottomed microtitre 96 well plates as described by [33,34] with some modifications. Briefly, 100 µL of overnight culture of *P. aeruginosa* (inoculum size was 1 × 10^8^ CFU/mL) was added to each well of the plates in the presence of 100 µL subinhibitory concentrations (subMIC) of extract and oil (0.5, 0.25 and 0.125 MIC) or 100 mL medium (control). After incubation for 24 h at 37 °C, each well was washed twice with sterile PBS (pH 7.4), dried, stained for 10 min with 0.1% crystal violet in order to determine the biofilm mass. After drying, 200 µL of 95% ethanol (*v/v*) was added to solubilize the dye that had stained the biofilm cells. The excess stain was washed off with dH_2_O. After 10 min, the content of the wells was homogenized and the absorbance at λ = 625 nm was read on a Sunrise™—Tecan ELISA reader (Mannedorf, Switzerland). The experiment was done in triplicate and repeated two times and values were presented as a mean values ± SE.

### 3.4. Inhibition of Biofilm Formation of P. aeruginosa

*P. aeruginosa* was cultured overnight at 37 °C in LB medium and adjusted to a concentration of 1.0 × 10^8^ CFU/mL for final inoculum. Filter paper discs (Whatman; 4 mm in diamater) were impregnated with a solution of *A. blazei* extract (0.125, 0.25, 0.5 mg/disc), streptomycin and ampicillin (0.125, 0.25, 0.5 mg/disc). Discs were dried at room temperature (3 h, protected from light), and aseptically placed onto the plates prior inoculated with *P. aeruginosa* (1 × 10^8^ CFU/mL). Petri dishes were placed for incubation in a thermostat at 37 °C for 24 h. After incubation, it was recorded whether inhibition or antiquorum zones were obtained. Minimal inhibitory concentrations were determined as a diameter of the growth clear inhibition zones around the discs (no growth at all), while antiqourum, *i.e.*, antibiofilm zones were determined as hazy zones around the discs (growth with different characteristics) behind the margin of the inhibition zone [35]. The experiment was done in triplicate and repeated two times and values were presented as mean values ± SE.

### 3.5. Inhibition of Twitching and Flagella Motility of P. aeruginosa

After growth in the presence or absence of *A. blazei* extract (subMIC 0.15 mg/mL), streptomycin and ampicillin (subMIC 0.15 mg/mL), the cells of *P. aeruginosa* PA01 were washed twice with sterile PBS and resuspended in PBS at 1 × 10^8^ cfu/mL (OD of 0.1 at 660 nm). Briefly, cells were stabbed into a nutrient agar plate with a sterile toothpick and incubated overnight at 37 °C. Plates were then removed from the incubator and incubated at room temperature for two more days. Colony edges and the zone of motility were measured with a light microscope [28,29]. Fifty microlitres of *A. blazei* extract was mixed into 10 mL of molten MH (Mueller-Hinton) agar medium and poured immediately over the surface of a solidified LB agar plate as an overlay. The plate was point inoculated with an overnight culture of PAO1 once the overlaid agar had solidified and incubated at 37 °C for 3 days. The extent of swimming was determined by measuring the area of the colony [35]. The experiment was done in triplicate and repeated two times. The colony diameters were measured three times in different direction and values were presented as mean values ± SE.

### 3.6. Inhibition of Synthesis of P. aeruginosa PA01 Pyocyanin

Overnight culture of *P. aeruginosa* PA01 was diluted to OD_600 nm_ 0.2. Then, *A. blazei* extract (250 μL, 0.125 MIC (0.025 mg/mL), 0.25 MIC (0.05 mg/mL) and 0.5 MIC (0.10 mg/mL) was added to *P. aeruginosa* (4.75 mL) and incubated at 37 °C for 24 h. The treated culture was extracted with chloroform (3 mL), followed by mixing the chloroform layer with 0.2 M HCl (1 mL). Absorbance of the extracted organic layer was measured at 520 nm using a Shimadzu UV1601 spectrophotometer (Kyoto, Japan) [36]. The experiment was done in triplicate and repeated two times. The values for OD were presented as mean values ± SE.

## 4. Conclusions

In summary, our study indicated that *A. blazei* hot water extract possesses anti-quorum sensing activity against *P. aeruginosa*. Inhibition of bacterial quorum sensing offers a new strategy for the treatment of bacterial infections. The anti-quorum sensing property of *Agaricus blazei* may play an important role in antibacterial activity and offers an additional strategy for fighting bacterial infections.
